# The Functional Significance of Chiral Genitalia: Patterns of Asymmetry, Functional Morphology and Mating Success in the Praying Mantis *Ciulfina baldersoni*

**DOI:** 10.1371/journal.pone.0128755

**Published:** 2015-06-24

**Authors:** Gregory I. Holwell, Olga Kazakova, Felicity Evans, James C. O’Hanlon, Katherine L. Barry

**Affiliations:** 1 School of Biological Sciences, The University of Auckland, Auckland, 1142, New Zealand; 2 Department of Biological Sciences, Macquarie University, Sydney, NSW 2109, Australia; University of Idaho, UNITED STATES

## Abstract

Genital asymmetry is relatively common and widespread throughout the animal kingdom. The functional significance of genital asymmetry is however, poorly understood for most species. Male praying mantids of the genus *Ciulfina* are remarkable in possessing complex and directionally asymmetric genital phallomeres in some species, and chirally dimorphic/antisymmetric genitalia in others. Here we explore the chiral dimorphism in male genitalia of *Ciulfina baldersoni* which appear to exhibit genital antisymmetry. We test whether genital orientation influences mating success, copulation duration and the attachment duration of spermatophores. Additionally we investigate genital interactions between male and females using x-ray micro-computed tomography (micro-CT) and scanning electron microscopy (SEM). Lastly we assess whether genital asymmetry is associated with non-genital morphological asymmetry of a range of traits. Our results highlight the complex functional morphology of genitalia in this praying mantis species and yet demonstrate no functional difference between dextral and sinistral morphs other than the direction of attachment with both morphs enjoying equal levels of mating success. Chiral morphs also did not strongly associate with any other forms of asymmetry. We therefore conclude that genital chirality in *Ciulfina baldersoni* is a likely case of antisymmetry with no functional significance to genital orientation, and is likely to be selectively neutral.

## Introduction

Morphological asymmetries arise in all organisms and can manifest in a variety of forms [1, 2]. Overt deviations are classed as either directional asymmetry or chiral asymmetry/antisymmetry. In cases of directional asymmetry, more than 95% of individuals display asymmetry towards their left (sinistral) or right (dextral) sides. Chiral asymmetry/antisymmetry involves populations containing roughly equal proportions of sinistral and dextral individuals [1]. The phenomenon of chirality occurs with most familiarity within individuals, as occurs with human hands, the left being the mirror image of the right. However, chirality can occur in whole regions of the body, whereby the direction of asymmetry can vary among individuals and they can be classified as either dextral or sinistral. This is particularly obvious in snails, where most of the body is coiled [3]. Some snail species are dextral, others are sinistral, and some exhibit variation in chiral frequencies both among and within populations.

One of the most vivid and least studied examples of asymmetry is seen in chirally asymmetric genitalia, where two mirror-image asymmetric genital morphs exist within one species [4, 5]. Genital chirality has so far been observed in at least 13 insect genera [5], but due to the common use of genital traits in taxonomy, many cases of chirality may have been classified as aberrations or new species [6]. In most known cases of genital antisymmetry, male genitalia are chiral, but female genitalia are symmetrical. Female genital chirality is less prevalent, and in these cases the male genitalia appear symmetrical. There are a number of hypotheses for the evolution of asymmetric genitalia from symmetric structures [6], but the adaptive significance of switches from directional asymmetry to antisymmetry is unclear [5]. As switches from dextrality to sinistrality (and vice versa) are known to have occurred in numerous taxa, it is possible that antisymmetry represents a phase along the path of such a switch [5]. Species in which antisymmetry appears to have reached a stable state are perhaps more perplexing.

The mantid genus *Ciulfina* offers one of the more intriguing cases of genital chirality available for examination. Of the eight species of *Ciulfina* for which genital morphology has been described, three possess chiral male genitalia, with inter-species and inter—population variation in the proportion of dextral and sinistral males [7–9]. However, the female genitalia of all species are symmetric [10–12]. Furthermore, four of the remaining species (*C*. *klassi*, *C*. *annecharlotteae*, *C*. *terrymariceae* and *C*. *herbersteinae*) possess directionally asymmetric, dextral male genitalia, with only one species (*C*. *ianrichardi*) possessing solely sinistral genitalia [7, 9, 12]. This is surprising because sinistral male genitalia are almost certainly plesiomorphic to the Mantodea, with only one other report of genital asymmetry reversal in the Cambodian mantid genus *Haania* [13]. Males of both genital orientations can mate with the same females [12]. During mating, the male twists his abdomen one way or the other (dextral males twist to the right, sinistral to the left), achieving intromission with the dorsal surface of the male genitalia contacting the ventral surface of the female genitalia [14] in a false-male-on-top position *sensu* [6]. The way in which male and female genital structures interact once copulation commences in *Ciulfina* is unstudied.

This study addresses three aspects of genital chirality in the chirally dimorphic praying mantis, *Ciulfina baldersoni*. Firstly, we conduct a series of double mating trials to assess whether genital chirality affects aspects of mating behaviour including copulation duration and spermatophore attachment. Secondly, we utilize micro-computed tomography and scanning electron microscopy to examine the interaction of male and female genitalia *in copula* and assess (a) whether the two genital morphs differ qualitatively in their functional morphology, and (b) the utility of these methods to generate hypotheses about the potential mechanisms driving the evolution of male genitalia in *Ciulfina*. Lastly, we assess whether the two morphs differ in non-genital trait size and the extent of non-genital asymmetry, and whether it corresponds with the genital phenotype.

## Methods

### Study species

The genus *Ciulfina* (Mantodea: Liturgusidae) encompasses a number of small, arboreal mantid species that occur along the north east coast of Australia [15–17]. These species are almost indistinguishable based on external body morphology but can be differentiated via detailed examination of male genital morphology [7]. The species selected for this study, *C*. *baldersoni*, occurs in the Hervey Bay and Bundaberg areas (Queensland, Australia) in small populations with limited dispersal. Individuals of both sexes are encountered mainly on narrow, smooth-barked trees with few branches [15].

Male *C*. *baldersoni* are characterised by possessing complex multi-component male genitalia that are extremely asymmetrical, existing in one of two chiral morphs [12], while female genitalia are symmetrical. Male Mantodean genitalia comprise a right phallomere (RP) and a left complex comprising dorsal (DLC) and ventral components (VLC) [18]. The most variable and species-specific elements of this genital complex are the distal process of the DLC or *processo apicale* (PAA), the distal process of the VLC or *processo distale* (PDA), and the ventral sclerotised process (VSP) of the RP. Dextral and sinistral males are approximately equally represented in these populations (Hervey Bay: 54% sinistral, 46% dextral; Bundaberg: 50% sinistral, 50% dextral) [12] and both morphs occupy the same habitat.

### Collection and husbandry

Subadult *Ciulfina baldersoni* were collected by hand from tree trunks around Hervey Bay and Bundaberg, Queensland, Australia and were housed in 50cm × 50cm × 40cm enclosures until adulthood. This species is not endangered or protected, and permission to collect was not required for any of our collection locations, as they were on public but unprotected land. Enclosures for housing the nymphs were constructed from flyscreen on wooden frames and fitted with vertical wooden sticks to mimic the mantids’ natural habitat. The base of each enclosure was lined with coco-peat, a commercially available product made from coconut husks, which was moistened daily to ensure adequate humidity for moulting. Nymphs were misted with water daily and supplied with *Drosophila melanogaster ad libitum* as a food source. Upon final eclosure, adults were separated into individual upturned plastic cups (10cm height, 4cm radius; with a mesh window for ventilation and a bark perch) and fed every 1–2 days with Queensland fruit flies (*Bactrocera tryoni*). All mantids were kept on a diurnal period of 10–12 hours of light per day and at a temperature of 25–30°C mimicking their natural conditions.

### Mating success, copulation duration and spermatophore attachment

We tested whether genital orientation (left/sinistral or right/dextral) affected male mating success using randomised double mating trials. This allowed us to assess relative male mating success, copulation duration and spermatophore attachment time among the first males to mate, and then subsequently assess these factors when females were paired with a second male. Females were initially paired with either a sinistral (n = 20) or dextral (n = 19) male and no males were reused for second matings. Females were therefore paired with two males in one of four treatments: 1) first a sinistral male followed by a dextral male, 2) first a dextral male followed by a sinistral male, 3) two sinistral males, and 4) two dextral males. The mating arena consisted of a smooth-barked tree trunk (60cm height, 25 cm diameter), sawn in half and attached to a perspex board to prevent mantids from moving out of view of the observer (following methods in [14]). A stopwatch was used to measure the time taken to mount, the duration of copulatory connection (from genital contact till dismount) and the time taken for the female to remove and fully consume the spermatophore after the male dismounted the female. Pronotum length was included in these analyses to allow us to assess the affect of size on copulatory behaviour (see below). To examine differences between sinistral and dextral males in behaviour exhibited during copulation with virgin and mated females, we used independent sample t-tests and ANOVA. To assess the relationship between pronotum length and behavioural data, we used Pearson’s correlations. All continuous data (size and behavioural data) were checked for normal distribution via the Kolmogorov-Smirnov test and all were found to be normally distributed (P > 0.05). Data were analysed using SPSS 17.0.

### Functional morphology

Twelve randomly chosen adult female *C*. *baldersoni* were paired with either a dextral (n = 6) or a sinistral (n = 6) male. After ten minutes of copulation the attached pair was asphyxiated with carbon dioxide. Initial exploratory work determined that this approach disturbed the pairs much less than immersion in liquid nitrogen, as the carbon dioxide could be administered via a tube to asphyxiate them while they remained attached to the tree trunk. Six pairs were fixed for SEM (methods follow [7]). A further six pairs were immersed in 70% ethanol for microCT. These were inserted in plastic tubing (diameter 10mm) sealed with dental wax to minimise desiccation. Each tube was mounted on a 10mm stub in the Skyscan 1172 (software version 1.4) and scanned (voltage: 40kV; current: 250uA) every 0.20° rotated about the vertical axis 360° with 590ms exposures creating images with a pixel size of 6.06μm. Images were reconstructed using NRecon version 1.4.4. Reconstructed files were archived in bitmap format, analysed and captured using VGStudioMax version 1.2. Completed 3D reconstructions were rotated 360° and multiple cross-sections were taken through each specimen at various angles and captured.

### Assessment of non-genital asymmetry

A total of 62 adult males were examined under a dissection microscope (Olympus SZX9) and scored twice for each of the following categorical traits: (1) genital orientation (left/right), (2) direction of folding of tegmina (left fore-wing over right or right fore-wing over left wing), (3) presence or absence of a secondary fork in the second vannal vein of the hind wings and (4) the number of spines on the internal sections of each raptorial limb (the internal femoral and tibial spines). Seven males had damaged or missing arms, leaving 56 males for analysis of spines (37 sinistral and 19 dextral). We defined internal femoral spines as all ventral (internal during “praying” position) spines of the femora including the terminal spine and excluding the discoidal spines. Internal tibial spines were defined as all ventral (internal during “praying” position) spines of the tibia including the terminal spine. Individuals displaying internal femoral spine (IFS) or internal tibial spine (ITS) symmetry were defined as displaying the same number of spines on the left and right raptorial femora/tibiae, while asymmetric individuals differed in the number of spines between the left and right raptorial femora/tibiae. Asymmetric venation in hind wing morphology was defined as the occurrence of a secondary fork in the second vannal vein of the left hind wing and no occurrence of a similar fork in the right hind wing, or vice versa. An individual was deemed to have symmetric venation if the fork occurred either in both hind wings, or neither. These traits were characterised for 37 sinistral and 20 dextral males. To determine whether sinistral or dextral males differed for these non-continuous variables we used Fisher’s exact tests.

### Bilateral asymmetry of continuous non-genital traits

Measurements of continuous size traits (total of 20 traits) on the left and right sides of the body were taken using a QImaging Micropublisher 5.0 camera mounted on an Olympus SZX9 dissecting microscope, with the aid of the QCapture Pro software. All images were processed using ImageJ software. Points of measurement on the tegmina were defined after examining multiple wings and are based on consistent points of venation and folding, approximating the length and width of the tegmina. On the raptorial arm we measured the ventral length of the coxa, femur, tibia and tarsus. Additionally we measured the length of the pronotum, and the femur and tibia length of each of the left and right meta- and mesothoracic limbs (hind- and mid-legs) (Table 1). To determine if there was a difference in the degree of asymmetry in continuous traits between dextral and sinistral males, we measured the level and direction of asymmetry of each continuous trait within an individual by subtracting the right variable from the left variable. Data were analysed using SPSS 17.0. All continuous data were tested for normality and none deviated significantly from a normal distribution (Kolmogorov-Smirnov, all P > 0.05).

**Table 1:** Summary of means and standard deviations of 24 morphological measurements for males of sinistral and dextral genital orientation.

	Measurement*	Mean	Standard Deviation		Measurement*	Mean	Standard Deviation
Sinistral	L wing 1	3.7095	0.2139756	Dextral	L wing 1	3.773214	0.2638287
	L wing 2	12.149542	0.3992031		L wing 2	12.187786	0.3025457
	R wing 1	3.702667	0.2198651		R wing 1	3.812714	0.2985146
	R wing 2	12.173417	0.4032213		R wing 2	12.187786	0.3108804
	L raptorial 1	4.675304	0.1513638		L raptorial 1	4.645864	0.1713704
	L raptorial 2	6.201336	0.1889309		L raptorial 2	6.227013	0.1605485
	L raptorial 3	2.714129	0.1166768		L raptorial 3	2.748944	0.0957999
	L raptorial 4	1.060898	0.1081202		L raptorial 4	1.061011	0.1113919
	L midleg 1	8.45528	0.2676965		L midleg 1	8.472826	0.1949813
	L midleg 2	6.518628	0.2192132		L midleg 2	6.624749	0.1841248
	L hindleg 1	9.098541	0.3277344		L hindleg 1	9.167898	0.2408988
	L hindleg 2	9.702412	0.368322		L hindleg 2	9.894368	0.2813689
	R raptorial 1	4.682332	0.1252823		R raptorial 1	4.695777	0.1237778
	R raptorial 2	6.192227	0.2314822		R raptorial 2	6.213909	0.171212
	R raptorial 3	2.727684	0.1083443		R raptorial 3	2.761713	0.089839
	R raptorial 4	1.057544	0.0725484		R raptorial 4	1.046638	0.1542123
	R midleg 1	8.434633	0.2944969		R midleg 1	8.472077	0.2289311
	R midleg 2	6.529635	0.2436073		R midleg 2	6.681008	0.2209303
	R hindleg 1	9.083667	0.2995231		R hindleg 1	9.133734	0.2600706
	R hindleg 2	9.651321	0.4148491		R hindleg 2	9.845898	0.2526999
	ITS (L)	9.041667	0.3586408		ITS (L)	9	0
	IFS (L)	13.833333	0.3806935		IFS (L)	13.785714	0.4258153
	ITS (R)	9	0.2948839		ITS (R)	9.071429	0.2672612
	IFS (R)	13.708333	0.6902531		IFS (R)	13.642857	0.6333237

*Measurement names correspond to those described in the Methods section. Values are in millimetres.

### Measurement error

To calculate measurement error [19] we twice measured the traits on the right hand sides of ten randomly chosen males. Measurements were taken 48 hours apart and the order of males was randomised before the second measurement was taken. We then calculated ME1 (Measurement Error; [19]) by first subtracting each measurement taken on the first day from the ones taken on the second day. Then the averages of the absolutes of these values for each trait were calculated $ME1=\frac{\sum \left[M1-M2\right]}{N}$ where M1 and M2 are the measurements taken on the first and second day and N represents the number of individuals in which this trait was measured). The means of trait asymmetry for each variable were compared with the ME for that variable, and the %ME calculated. If the %ME was less than 100% we interpreted the remaining variation as measurable asymmetry. To determine the repeatability of our measurements, we calculated the regression values from the two days’ data. Traits were used in further analyses if our measure of them was highly repeatable (R^2^ ≥ 0.90). As a consequence, from the original ten non-genital size traits, we only considered six traits in our subsequent analyses (see Table 2

**Table 2:** Summary of means and standard deviations (SD) of the degree of asymmetry for 12 paired morphological measurements for males of sinistral and dextral genital orientation (refer to methods for calculation of degree of asymmetry).

	Measurement*	Mean	SD		Measurement*	Mean	SD
Sinistral	wing 1	0.0068	0.12073	Dextral	awing 1	-0.0395	0.18542
	wing 2	-0.0239	0.11772		wing 2	0	0.1474
	raptorial 1	-0.007	0.09938		raptorial 1	-0.0499	0.12356
	raptorial 2	0.0091	0.09047		raptorial 2	0.0131	0.06954
	raptorial 3	-0.0136	0.09961		raptorial 3	-0.0128	0.0806
	raptorial 4	0.0034	0.08131		raptorial 4	0.0144	0.09384
	midleg 1	0.0206	0.10425		midleg 1	0.0007	0.06881
	midleg 2	-0.011	0.09679		midleg 2	-0.0563	0.12331
	hindleg 1	0.0149	0.09289		hindleg 1	0.0342	0.06868
	hindleg 2	0.0511	0.14444		hindleg 2	0.0485	0.11384
	ITS	0.0417	0.46431		ITS	-0.0714	0.26726
	IFS	0.125	0.67967		IFS	0.1429	0.77033

*Measurement names correspond to those described in the Methods section. Values are in millimetres.

## Results

### Behavioural differences between sinistral and dextral males: initial copulation

**Fig 1:**
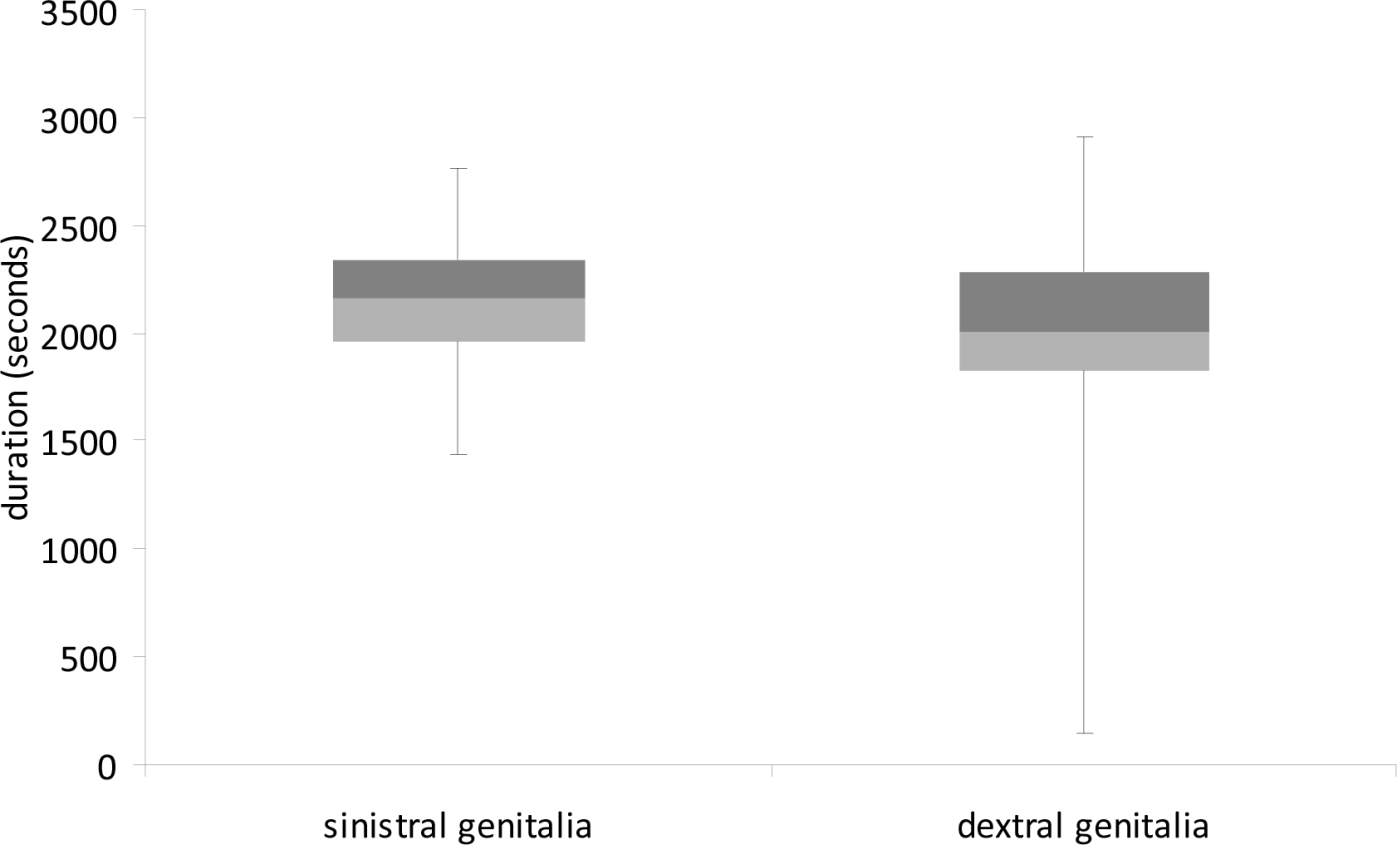
Copulation duration (seconds) of *C*. *baldersoni* males with sinistral and dextral genitalia when mated with virgin females. Boxplots show medians and first and third inter-quartile ranges. Whiskers are extended to maximum and minimum values.

**Fig 2:**
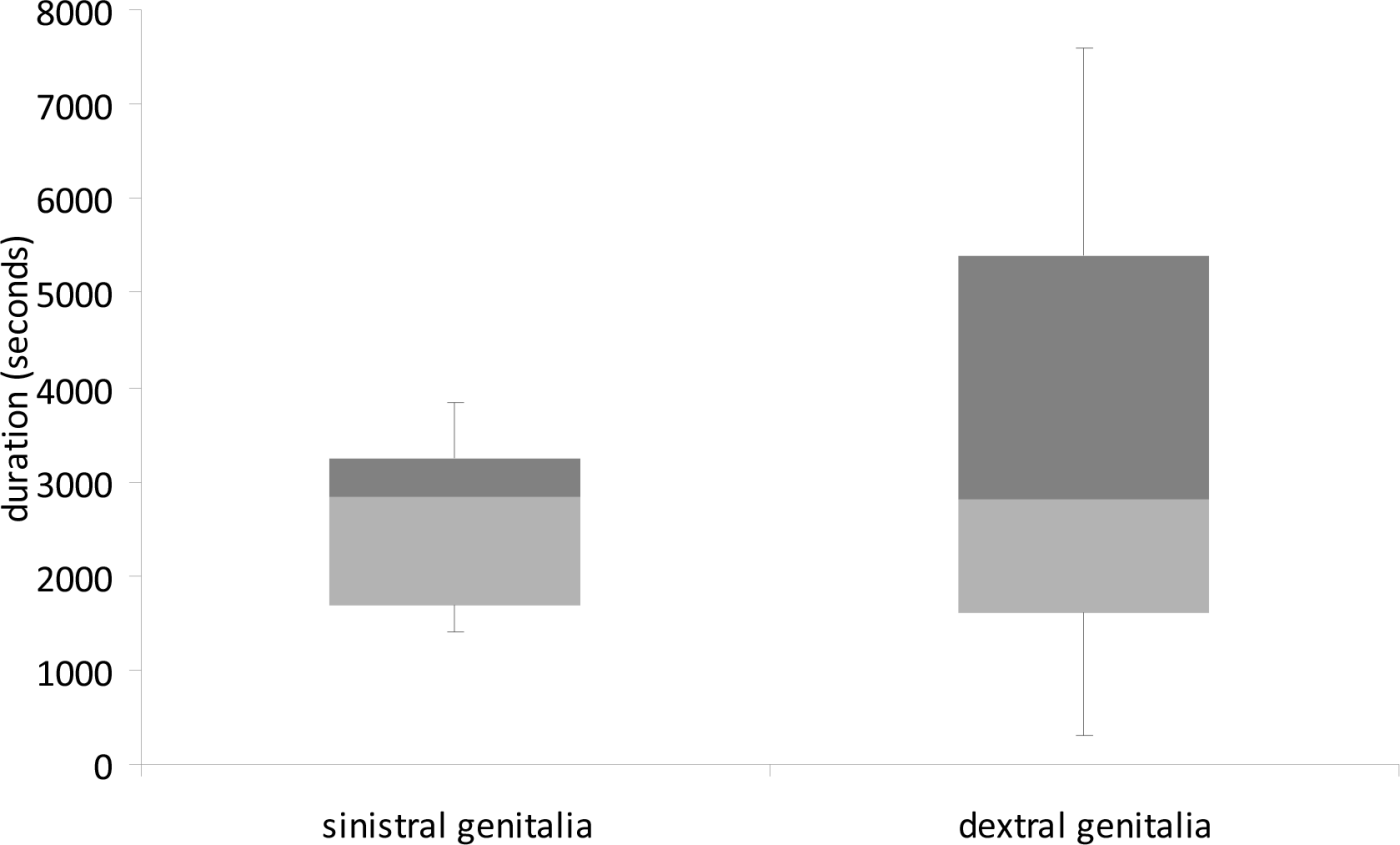
Post-copulatory spermatophore attachment duration (seconds) of *C*. *baldersoni* males with sinistral and dextral genitalia when mated with virgin females. Boxplots show medians and first and third inter-quartile ranges. Whiskers are extended to maximum and minimum values.

Although we found no significant difference in mean copulation duration of dextral (n = 19) and sinistral (n = 20) males (Independent samples t-test: t = 1.042, df = 38, P = 0.304; Fig 1) or postcopulatory spermatophore attachment time (Independent samples t-test: t = -1.328, df = 16.35, P = 0.202; Fig 2), Levene’s test revealed greatly unequal variances between dextral and sinistral males in the duration of post-copulatory spermatophore attachment time (Levene’s test: F = 20.053, P < 0.001). Specifically, postcopulatory spermatophore attachment time was much more variable for dextral males than for sinistral males.

**Fig 3:**
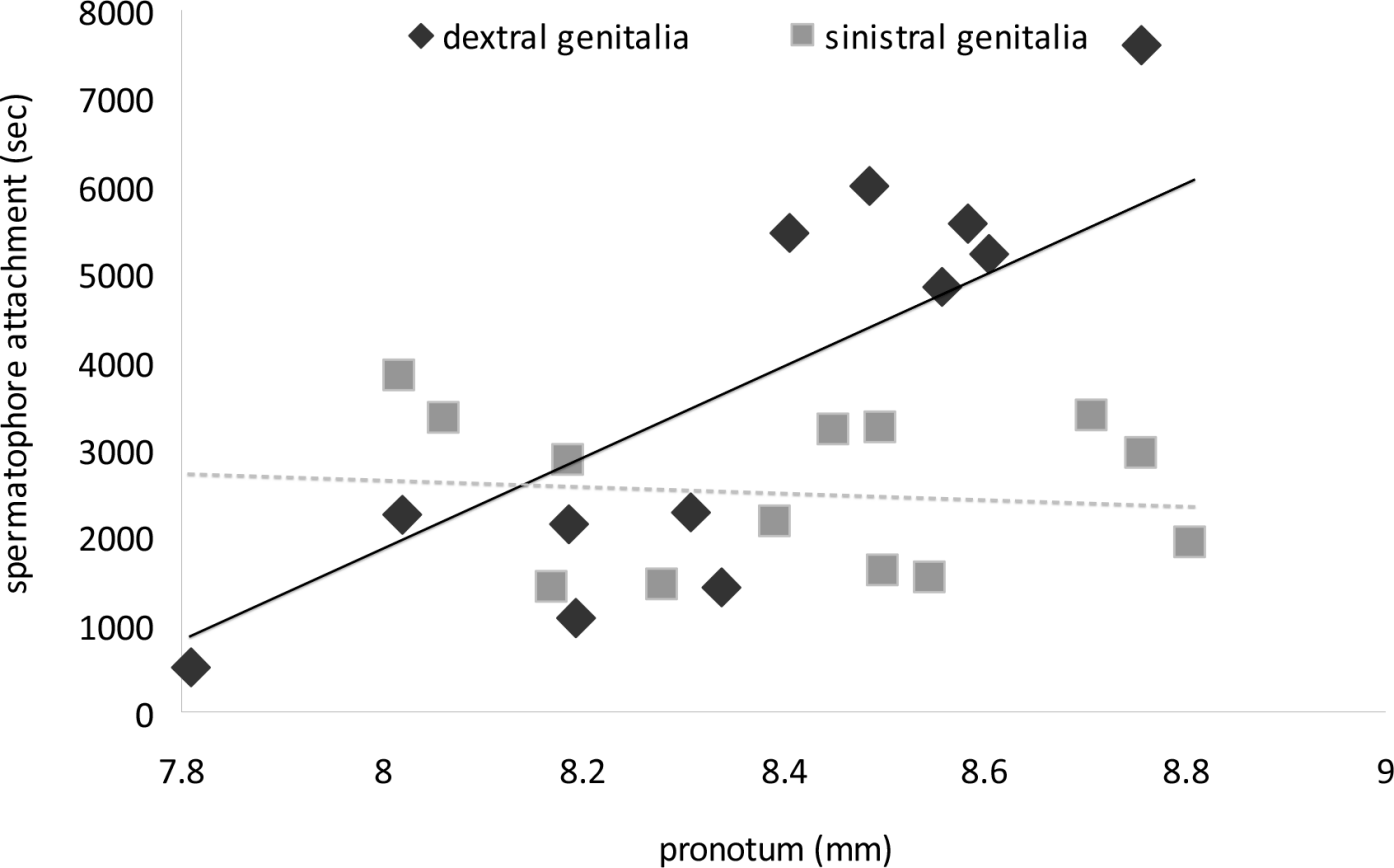
Relationship between male pronotum length (mm) and the duration of post-copulatory spermatophore attachment for sinistral (grey squares and dashed grey trendline: slope (y) = -537x; Pearson correlation: r = -0.159, n = 13, P = 0.604) and dextral (black diamonds and solid black trendline: slope (y) = 4989x; Pearson correlation: r = 0.582, n = 13, P = 0.037) male *C*. *baldersoni*.

Additionally, postcopulatory spermatophore attachment time was positively correlated with male pronotum length for dextral males (Pearson correlation: r = 0.582, n = 13, P = 0.037) but not for sinistral males (Pearson correlation: r = -0.159, n = 13, P = 0.604)(Fig 3).

### Behavioural differences between sinistral and dextral males: second copulation

We found no significant effect of double mating treatments (Left/Left, Left/Right, Right/Left and Right/Right) on the copulation duration and post-copulatory spermatophore attachment time of the second male to mate (One-way ANOVA: pronotum: F_3, 22_ = 0.698, P = 0.563; copulation duration: F_3, 27_ = 0.488, P = 0.693; spermatophore attachment: F_3, 23_ = 1.282, P = 0.304).

### Scanning electron microscopy

**Fig 4:**
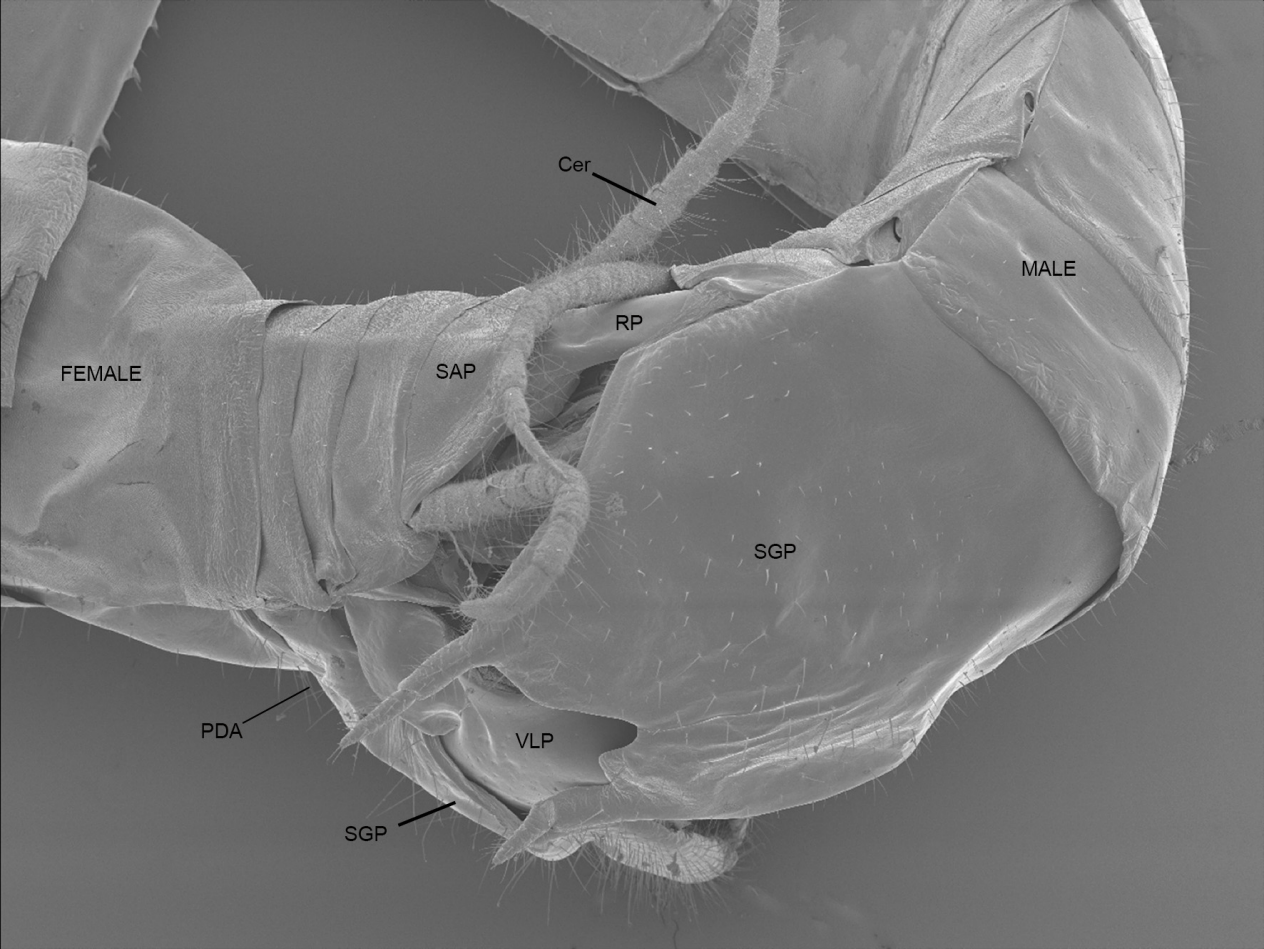
Scanning electron micrograph showing external interaction between male and female *C*. *baldersoni* genitalia in copula. Cer = cercus; PDA = processo distale (position of spines can be inferred from distention of female abdomen); RP = right phallomere; SAP = supra-anal plate; SGP = sub-genital plate; Sp = external tip of spermatophore.

SEMs of mating pairs demonstrate the arrangement and interaction of only some male and female structures during copulation (Fig 4). The positions of male and female subgenital and supra-anal plates are observable as are most of the male VLC and RP (Fig 4). The position of the jagged PDA can be inferred from the distention of the female abdomen (Fig 4). The positions of male structures upon the bilaterally symmetrical female genitalia are identical but mirror-imaged between sinistral and dextral males.

### Tomography

**Fig 5:**
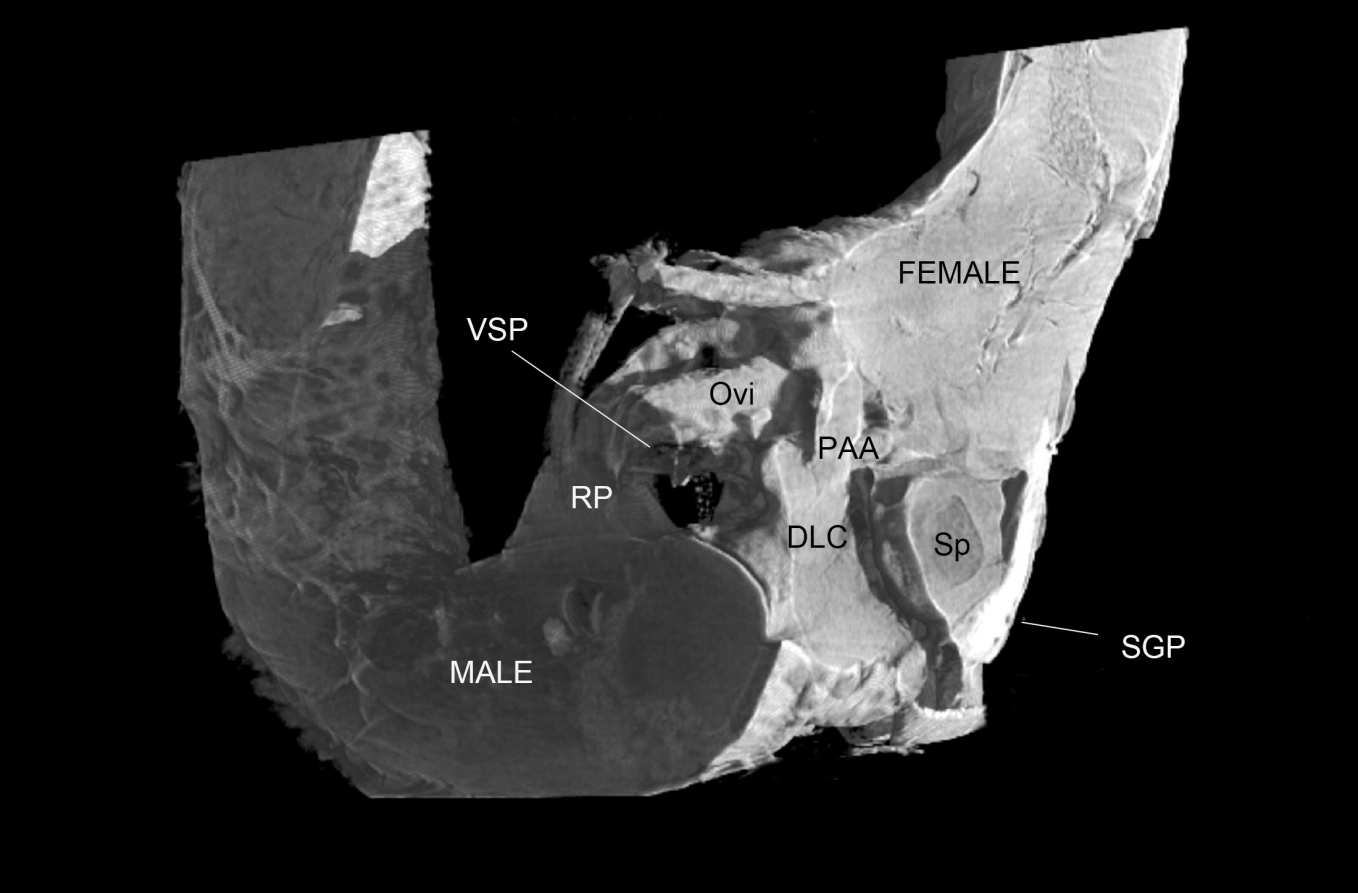
microCT 3D reconstructed image of interacting male and female genitalia sectioned along two planes. DLC = dorsal left complex; Ovi = ovipositor; PAA = processo apicale; RP = right phallomere; SGP = sub-genital plate; Sp = spermatophore; VSP = ventral sclerotised process

For observation of external genital interactions, micro computed tomographs were congruent with scanning electron micrographs. MicroCT was invaluable in discerning the positioning of internal genital structures and those concealed by the subgenital plates. The PDA, VSP and the entire DLC along with the spermatophore were not observable using SEM, but their placement within the female became apparent through the use of microCT. (Fig 5).

### Interaction between male and female genitalia

Both the RP and VLC appear to serve the function of splaying open the female genitalia and anchoring the male in position. The wide plate of the VLC, when twisted, props open the female sub-genital plate and supra-anal plate. The jagged and highly sclerotised PDA is positioned against the dorsal proximal surface of the female subgenital plate. The female ovipositor complex is gripped between the VSP and FDA of the RP. The DLC is exerted into the female vestibulum (Fig 5). The dorsal edge of the exerted DLC presses against the ventral surface of the ventral valves of the ovipositor, while the ventral surface of the DLC presses against the proximal surface of the female subgenital plate anterior to the male VLC, thus sealing the vestibulum. The spermatophore remained relatively undamaged during microCT processing. Fig 5 shows that the spermatophore is inserted deeply into the female genital cavity during copulation, as far as the interface between the vestibulum and the genital chamber. The spermatophore is supported by the PAA, which is inserted ventrally to the ovipositor within the female (Fig 5). The orientation of dextral male genitalia within the female was observed to be a mirror-image of the orientation of sinistral male genitalia within the female.

### Non-genital trait size in sinistral and dextral males

We did not detect any significant differences between the 24 non-genital morphological measurements between dextral and sinistral males (all >0.1; Table 1). As these results were clearly non-significant, there was no reason to account for an increased probability of type-1 statistical errors when performing multiple tests, such as a test for false discovery rate or Bonferroni correction. Similarly, as this exploratory analysis identified no differences for any trait, combined multivariate analysis was deemed to be unnecessary.

### Comparing trait asymmetry in sinistral and dextral males

Similarly, we found no difference between the level of absolute asymmetry between all 12 traits measured for dextral and sinistral males (all >0.1; Table 2). Comparing the absolute asymmetry of the sinistral and dextral males gives an indication of how high/low the fluctuating asymmetry is for each male type in relation to the other. Therefore, our results indicate that males possessing sinistral or dextral genitalia have similar levels of fluctuating asymmetry in non-genital traits.

### Asymmetry of wing folding, wing venation & spine asymmetry

The directionality of tegminal folding showed a marginal difference between dextral and sinistral males. Sinistral males preferentially folded their wings right over left (n = 22/33) while dextral males preferentially folded their wings left over right (n = 13/21) (Fisher’s exact: P = 0.052). Although there were a higher proportion of sinistral males displaying asymmetric venation of the hind wings (49% of sinistral males showed anal vein asymmetry), this proportion was not significantly different from dextral males (35% asymmetry; Fisher’s exact: P = 0.406). There was no statistically significant difference observed between sinistral and dextral males in either the femoral (Fisher’s exact: P = 0.777) or tibial (Fisher’s exact: P = 0.146) spine counts.

## Discussion

We have demonstrated that for *Ciulfina baldersoni*, the functional significance of male chiral genital dimorphism is probably negligible, and a likely example of antisymmetry which is selectively neutral. Male genitalia of both morphs interact with female genitalia in essentially the same way, there is no association between the direction of male genital asymmetry and the extent of asymmetry of any non-genital traits examined, and genital orientation did not influence mating success, copulation duration or the duration of spermatophore attachment with either virgin or mated females. Intriguingly, dextral males exhibited greater variance in copulation duration and spermatophore attachment duration. Dextral males also showed a correlation between body size and spermatophore attachment duration, whereas sinistral males did not.

Despite the male dimorphism in *C*. *baldersoni*, there was no qualitative difference in the placement of genitalia during copulation between the two male morphs, aside from their left-right orientation. The latter also includes the twisting direction of the male abdomen when mounting the female: to the left in sinistral males and to the right in dextral males. We found no evidence for any functional difference in the interactions between females and dextral or sinistral males. This is likely attributable to the fact that female genitalia are symmetrical [7,10]. Functional differences between chiral morphs during mating may be more likely in species where both males and females exhibit structural chirality. For example, both male and female high-spired snails (*Partula suturalis*) exhibit chirally dimorphic coiling. This species is capable of interchiral copulation, attributed to variable mounting orientation, but intrachiral matings occur more easily [3]. Conversely, in another snail, *Amphidromus inversus*, interchiral matings are functionally different and spermatophore transfer occurs more frequently for interchiral matings [20].

We did not find a strong connection between genital chirality and any of the non-genital features examined. Specifically, the direction of genital asymmetry did not have a statistically significant effect on the size or asymmetry of the walking legs, the raptorial spines or the wings. These data suggest that genital chirality in the genus *Ciulfina* is not linked to or influenced by non-genital morphological variation. These results are in line with studies of other taxa (Cockroaches: *Phyllodromica montana* [21], Sericini chafers [22] where variation in the direction of genital asymmetry was not associated with variation in the direction of asymmetry of non-genital traits. Sinistral and dextral male *C*. *baldersoni* did not differ significantly in the size of any non-genital traits and males possessing sinistral or dextral genitalia were found to have similar levels of fluctuating asymmetry. The direction of the genitalia did not affect the general balance of asymmetry in the body in either dextral or sinistral males. Indeed, asymmetries of all non-genital traits were confirmed to be non-directional fluctuating asymmetries (unimodally distributed), as opposed to chiral asymmetries (bimodally distributed). Our results suggest that the dramatic differences between sinistral and dextral genitalia do not extend to the legs and wings. Retournement of male genitalia over time (progressive slow stages of twisting) would be more likely to strongly affect body asymmetries than a mutation of a gene involved in early development, which can cause a switch in the position of physical features without affecting other areas [6]. However, investigation into the asymmetry of internal genital structures would be required to completely rule out retournement. As for many species exhibiting asymmetric genitalia, the morphological structure of asymmetric genital components appears to be linked to mating position [6], which in *Ciulfina* is also asymmetric [14]. *Ciulfina* males need to twist their posterior abdomen around substantially in order to connect with the female’s genitalia, but dextral and sinistral males do so equally efficiently, exactly mirroring each other in approach, mount and insertion.

Currently the most widely accepted explanation for the diversity and complexity of male genitalia is that sexual selection drives genital divergence among species [23–25]. This hypothesis incorporates three possible mechanisms: cryptic female choice, male-male competition and sexual conflict [25], which are difficult to discriminate and not mutually exclusive. In previous studies, the mechanisms of sexual selection involved in genital evolution have been greatly elucidated by *in situ* observations of genitalia during copulation. For example, recent examination of Sepsid flies revealed that male surstyli function in female stimulation, and not female restraint as previously thought based on morphology alone [26]. In contrast, functional morphological studies of the dung beetle, *Onthophagus taurus*, implicated both sperm competition and sexual conflict in driving the evolution of different male genital components [27].

Complications with studies of functional morphology arise in viewing internal interactions, retaining animals in copula and preventing distortion during histological preparation [28]. Our current study overcomes many issues by using non-invasive micro-CT imaging technology. X-ray computed tomography enables imaging of internal structures without the complications inherent in histological preparation and recent research has demonstrated the usefulness of micro-CT for investigating genital evolution in Arthropods [29, 30]. We have shown that micro-CT, particularly in conjunction with SEM is a powerful tool for the elucidation of internal interactions between male and female genitalia.

Our work on the functional morphology of genitalia in *C*. *baldersoni* suggests a number of testable hypotheses for the mechanisms driving genital evolution in *Ciulfina*. Sperm competition, sexual conflict and cryptic female choice are interrelated and unlikely to act in exclusion [27], and our direct observation of the positioning of each of the three major phallomeres suggests that different combinations of these mechanisms may be responsible for the individual and combined elaboration of these structures. Hosken *et al*. [28] observed incidental evidence of sperm competition acting on genitalia in male yellow dung flies (*Scathophaga stercoraria*), despite evidence of sexual conflict driving the evolution of other genital structures. Similarly, for *Onthophagus taurus*, different genital sclerites appear to have been shaped by sperm competition or sexual conflict [27]. In *Ciulfina klassi*, the shape of the DLC and the RP correlate with the number of the sperm transferred to the female spermatheca [31]. Here, our observations of the congeneric *C*. *baldersoni* help explain this association as the DLC appears involved in positioning the spermatophore. A direct role in sperm transfer suggests that sperm competition and selection for optimal spermatophore placement are likely mechanisms behind the evolution of DLC shape. We found no evidence that any genital structures reach the spermatheca and are involved in removal of rival sperm.

The structure and position of the PDA and VSP suggest a role for sexual conflict. Both structures are jagged and highly sclerotised, and are involved in gripping and anchoring to the female during copulation. Adaptations that allow males to grip onto females during copulation are among the classic examples of structures evolving through sexual conflict [32]. Potential tests of this hypothesis include ablation of these structures to investigate their role in copulation duration and/or overcoming female resistance, and comparison of the size/shape of the PDA and VSP of multiple *Ciulfina* species to investigate their relationship with female complicity and/or male attempts at usurpation. Sensilla on the ventral surface of the ventral valves of the ovipositor may suggest this to be a site of male stimulation, in accord with cryptic female choice. Indeed, during copulation, the male DLC is pressed against the ventral valves of the ovipositor and may stimulate female sensilla. The association between female sensory structures and potentially stimulatory male structures highlights the possibility of cryptic female choice in *C*. *baldersoni*. Experimental approaches involving ablation of male structures or desensitization of female sensilla, as described by Eberhard [33] would be useful in testing the cryptic female choice hypothesis. As different genital structures may be influenced by different mechanisms of sexual selection, further experimental and comparative work can now be targeted on the appropriate structures given our new understanding of how genitalia interact in *Ciulfina*.

Although we found no significant difference in mating success, spermatophore transfer success, copulation duration or the duration of postcopulatory spermatophore attachment, two aspects of these results are worthy of discussion. Firstly, dextral males were more variable in the duration of both copulation and postcopulatory spermatophore attachment. Secondly, dextral males showed a significant relationship between pronotum length (a proxy for body size) and spermatophore attachment duration. Dextral genitalia are more common among *Ciufina* species, and although sinistrality is a likely plesiomorphy for the Mantodea, dextrality is more likely to be the case for *Ciulfina*. Although highly speculative, these two results may suggest that females can respond to variation among dextral males, but not sinistral males. If the female genital structures that contact the male phallomeres were adapted to stimulation from dextral males due to internal sensory asymmetry, a recent switch to antisymmetry may mean that females are not sensitive to variation among sinistral males. If this were the case, cryptic female choice may explain the greater variance in time taken for females to remove spermatophores from dextral males, and the fact that spermatophore attachment duration was longer for larger males. If females could not sense sinistral genital variation, there may be nothing for them to exercise choice over. Although speculative, these results suggest potential avenues for assessing how switches in symmetry and the mechanisms of sexual selection involved in genital evolution can be combined.

## Supporting Information

S1 Table Raw data for all behavioural experiments (XLSX)

S2 Table Raw data for all morphological measurements (XLSX)
